# Supplementary figures and images for: Integrative Analysis of Gene Expression and miRNAs Reveal Biological Pathways Associated with Bud Paradormancy and Endodormancy in Grapevine
Source: Plants (Basel). 2021 Mar 31;10(4):669. doi: 10.3390/plants10040669 (PMC8067045; doi:10.3390/plants10040669)

Height

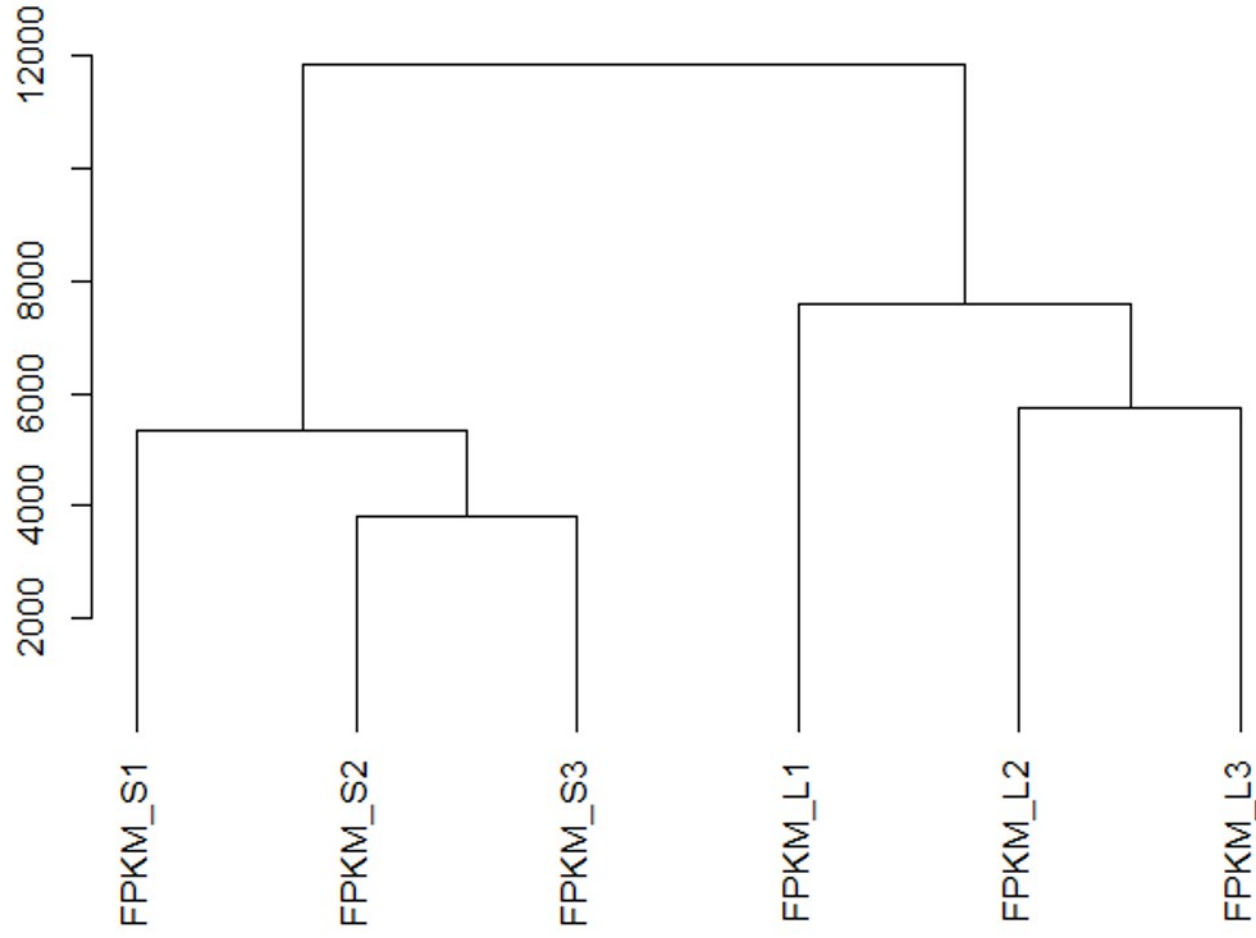

Supplement: Supplementary file 1 [file plants-10-00669-s001.zip › Figure S1.pdf]

$R^2 = 0.8$

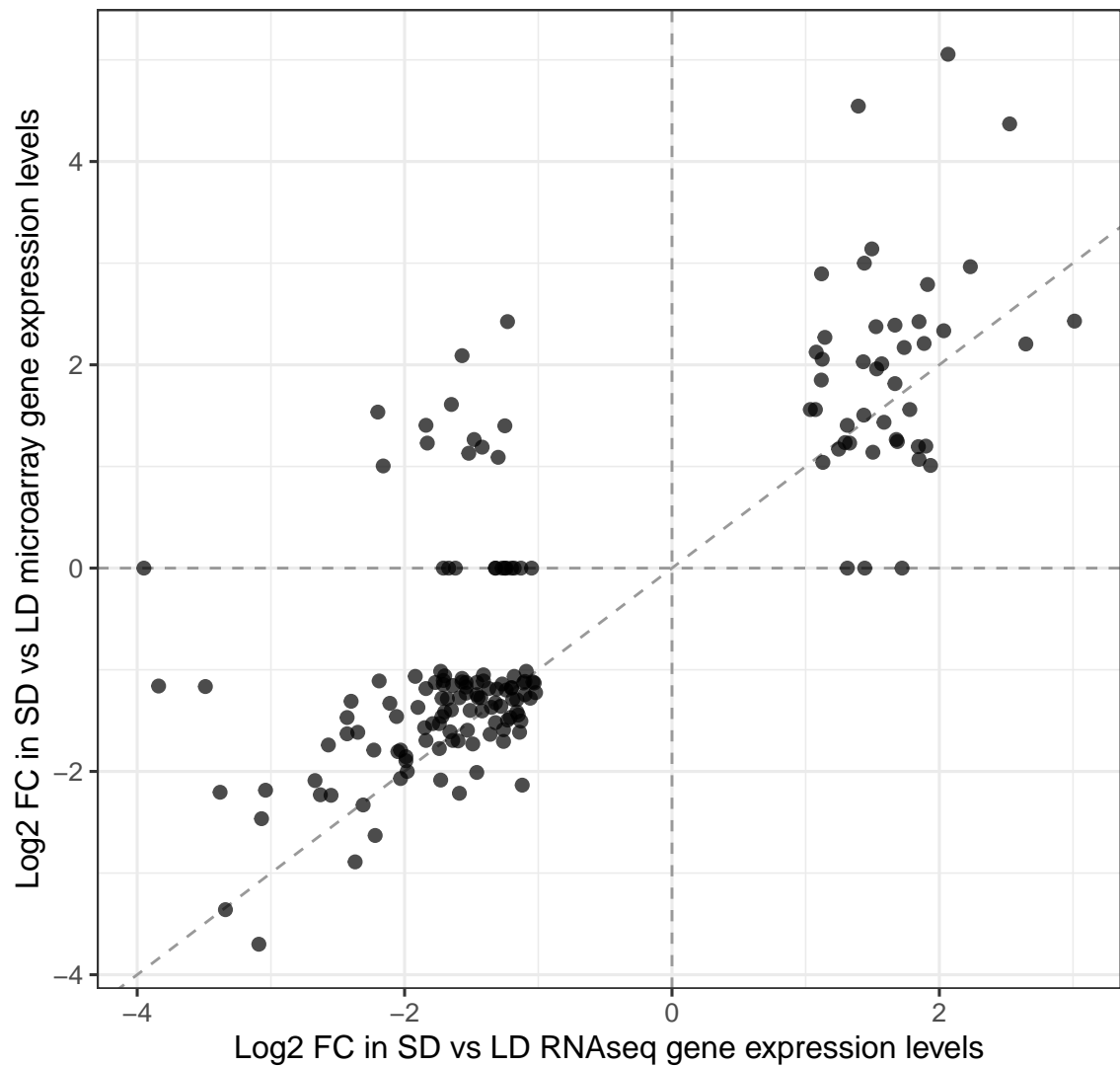

Supplement: Supplementary file 1 [file plants-10-00669-s001.zip › Figure S2_correlationPlot.pdf]

Number of sequences

LD 40,711  
SD 34,835

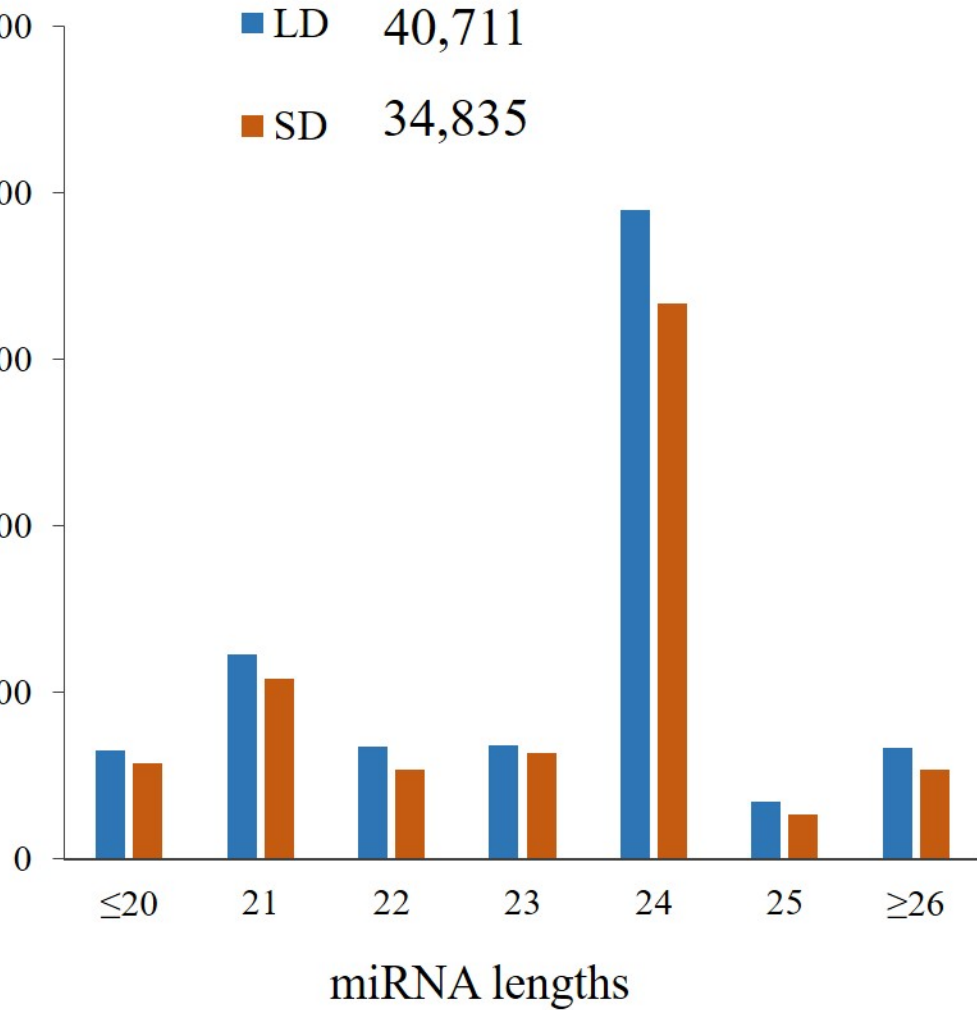

Supplement: Supplementary file 1 [file plants-10-00669-s001.zip › Figure S3a.pdf]

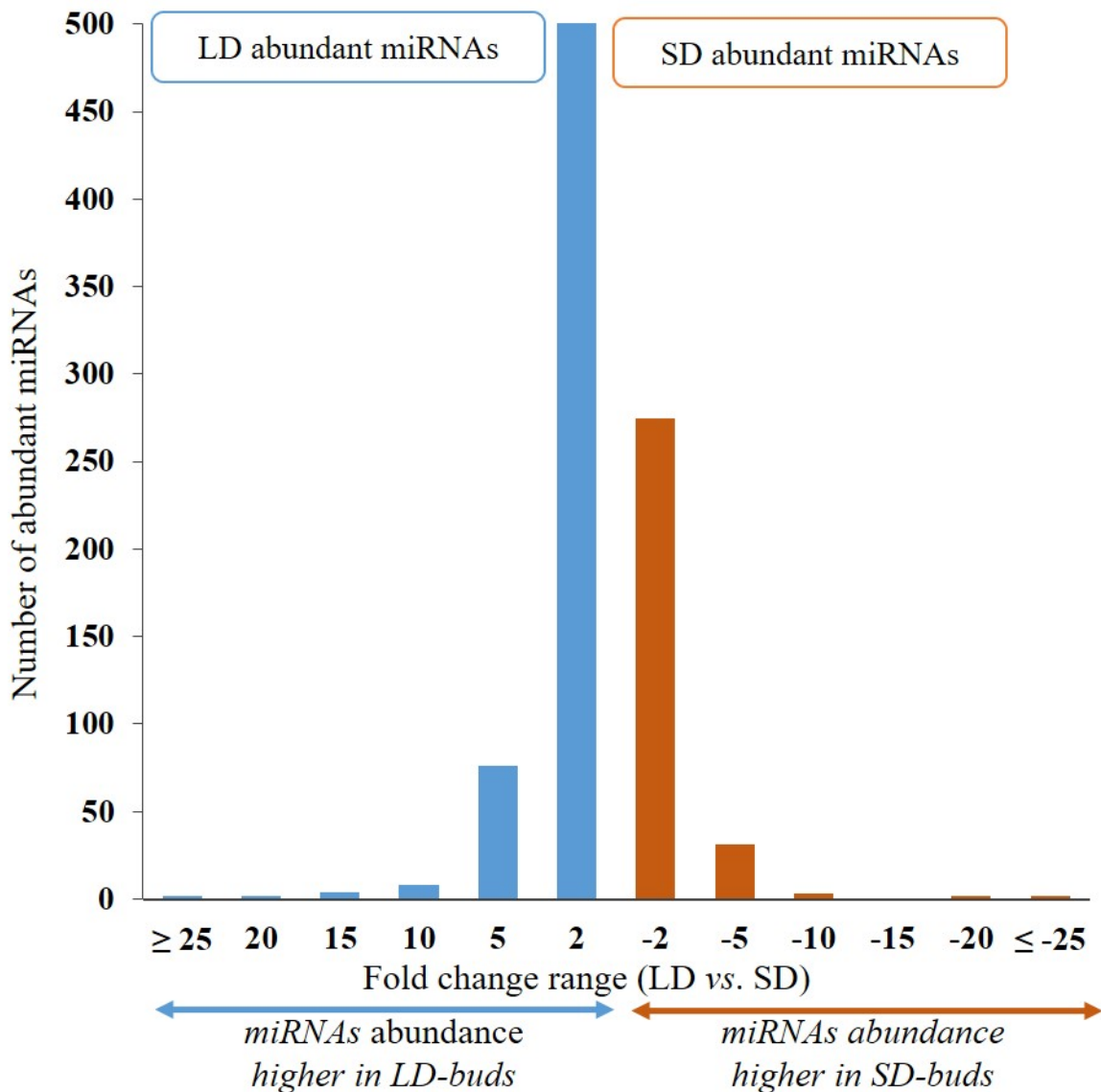

Supplement: Supplementary file 1 [file plants-10-00669-s001.zip › Figure S3b.pdf]

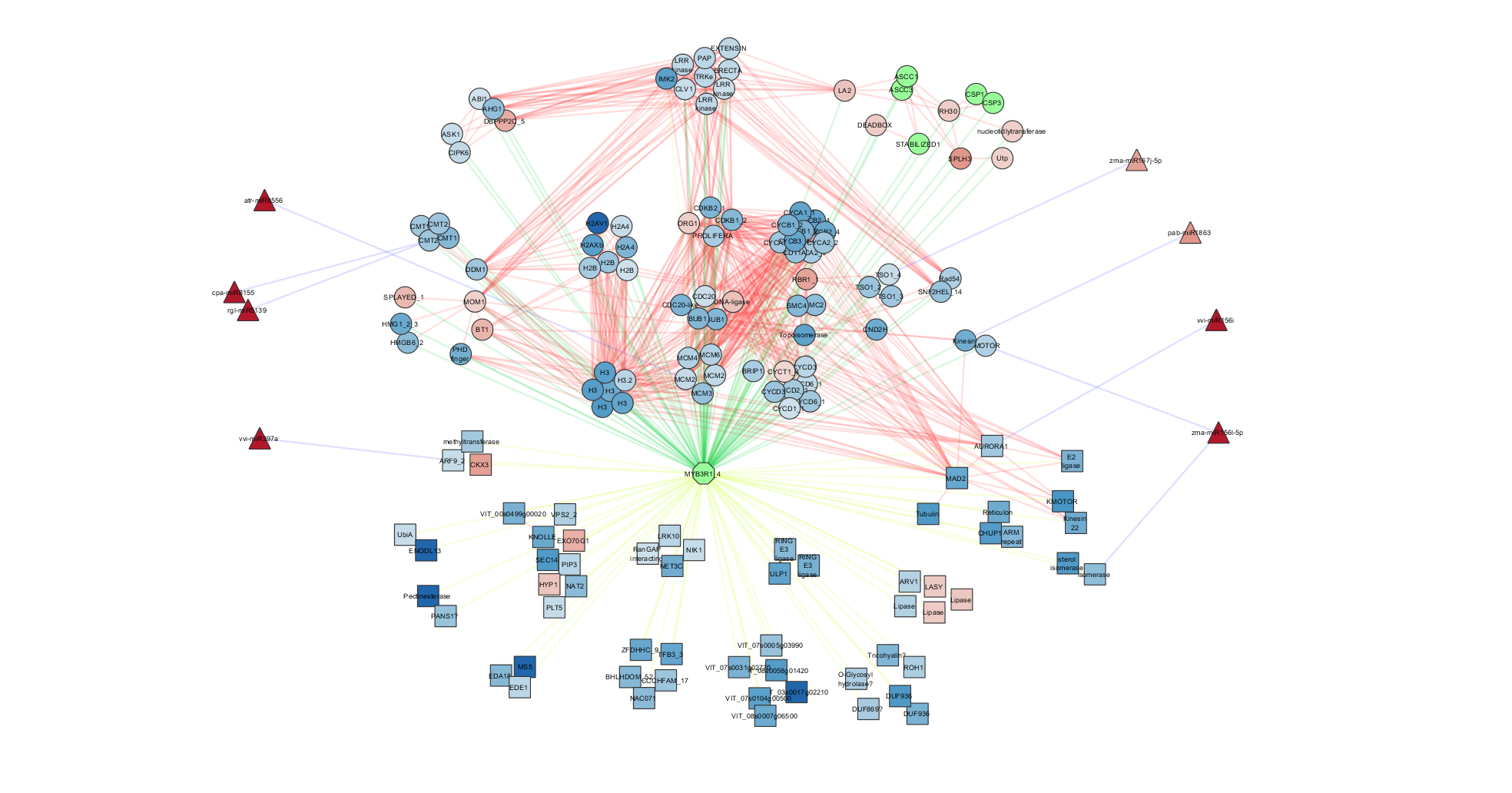

Supplement: Supplementary file 1 [file plants-10-00669-s001.zip › Figure4.png]
